# How academic achievement spreads: The role of distinct social networks in academic performance diffusion

**DOI:** 10.1371/journal.pone.0236737

**Published:** 2020-07-27

**Authors:** Sofia Dokuka, Diliara Valeeva, Maria Yudkevich

**Affiliations:** 1 Institute of Education, HSE University, Moscow, Russia; 2 Corpnet, Amsterdam Institute for Social Science Research, University of Amsterdam, Amsterdam, The Netherlands; 3 Center for Institutional Studies, HSE University, Moscow, Russia; Carlos III University of Madrid, SPAIN

## Abstract

Behavior diffusion through social networks is a key social process. It may be guided by various factors such as network topology, type of propagated behavior, and the strength of network connections. In this paper, we claim that the type of social interactions is also an important ingredient of behavioral diffusion. We examine the spread of academic achievements of first-year undergraduate students through friendship and study assistance networks, applying stochastic actor-oriented modeling. We show that informal social connections transmit performance while instrumental connections do not. The results highlight the importance of friendship in educational environments and contribute to debates on the behavior spread in social networks.

## Introduction

Social environment has a significant impact on individual decisions and behavior [1–3]. People tend to assimilate the behavior, social norms, and habits of their friends and peers. It is empirically shown that social interactions play a key role in the spread of innovations [4], health-related behavior [5,6], alcohol consumption and smoking [7,8], delinquent behavior [9,10], happiness [11], political views [12,13], cultural tastes [14], academic performance [15–19]. Although there is an extensive body of research showing that a large proportion of social practices disseminates across social networks [3], the question of what types of social contacts cause the spread of specific behavior remains open [1,20].

In this paper, we analyze the diffusion of academic performance across different types of student social networks. While these social networks are extensively studied in the literature [15–18,21,22], there is a lack of agreement on whether social networks are effective channels for the academic performance spread [16,18,23]. And if they are, what types of networks serve the best for the propagation of the academic-related behavior?

We analyze the spread of academic achievements within two different social networks of first-year undergraduate students. We test two mechanisms of academic performance diffusion in the student social networks. First, we analyze the academic performance spread through the *friendship network*, which can be considered as a network of informal social interactions. Second, we study the academic performance spread in the *study assistance network* which is aimed at study-related information and knowledge transmission, and serves for problem-solving [24]. We apply stochastic actor-oriented model (SAOM) for joint modeling of networks and behavior dynamics [25].

We model the evolution of two social systems. In the first model, we analyze the coevolution of friendship network and academic achievements. In the second model, we study the coevolution of study assistance network and academic achievements. Both models are controlled with a variety of structural and behavioral properties such as a tendency to form mutual ties and befriend similar others. Results show that academic performance spreads through friendship connections, while the study assistance ties do not cause the performance transfer.

### Literature review

Social networks are the pathways for the behavior transmission. This process may be guided by various factors, including the network topology [26], type of propagated behavior, nature of social contacts, and other features of the social environment. The majority of recent studies on this topic are concentrated on the structural properties of the networks that drive the behavior diffusion processes. For example, it was experimentally shown that short average path length and high clustering cause a faster behavior spread [27] that can be explained by the formation of the dense network communities with the fast information and behavior exchange within these cohesive groups. In [28] it was demonstrated that there are differences in spreading processes initiated by well-connected actors, or hubs, and by actors with a few social connections. Hubs are effective in information propagation due to the high number of connections, while actors with a few ties are more efficient in spreading messages that are controversial or costly. The probability of behavior adoption by an individual is also highly correlated with the number of social contacts that directly influence this individual [29]. The influence by many peers, or so-called “complex contagion”, results in faster and easier behavior adoption, rather than the influence by one person, or “simple contagion” [26]. The efficacy of social contagion is often associated with the type of propagated behavior. Centola and Macy outline the danger of considering the social contagion studies in the ‘whatever to be diffused’ way [30]. For example, the adoption of information is much less risky, costly, and time-consuming, rather than the adoption of health-related behavior, sports habits, and academic achievements.

The nature of social ties is also a significant factor for behavior transmission. Social connections are traditionally divided into “weak” and “strong” ties, and they exhibit completely different spreading patterns [31]. Strong ties are formed within the dense network communities such as family or friends, while weak ties, according to Granovetter’s definition, emerge during the whole life and represent people who are marginally included in the network of contacts, such as old college friends or colleagues [31]. Empirical literature shows that both types of relationships can serve as channels for the diffusion of behavior or information [1,20]. But weak ties are important instruments for information propagation, while strong ties are more successful in costly behavior transmission.

Although the vast array of theoretical and empirical studies improved our understanding of the behavior transmission processes, there is still an open question regarding the differences of the behavior spread in networks of different natures. Social ties can vary both in the level of their strength and intensity, as we outlined above, and in their origins. Networks can be based on friendship, romantics, advice seeking, social support, and many other relationships. Despite the huge variance in the social network types, the majority of the research on social diffusion is concentrated on the networks of friendship ties. However, the relationships of distinct nature can result in completely different behavior transmission processes.

In this paper, we consider the transmission of academic performance within student social networks. This process attracts the attention of researchers since the publication of the famous “Coleman Report” [21]. This report showed that students tend to obtain similar grades as their peers, classmates, and friends, and this effect remains strong after controlling for a variety of socio-economic and cognitive variables. Further empirical studies demonstrated the presence of this effect in various case studies. For example, it was shown that student grade point average (GPA) increases if her dormmate is in the highest 25th GPA percentile [32]. In [15], MBA students tend to assimilate the grades of their friends and advisers. It was also demonstrated that this social influence is associated with the personal characteristics of students and the nature of their social connections. For instance, lower-achieving students are more influenced by their peers [33,34], the diffusion of academic performance is stronger among women than men [35], can be related to the race of a peer [36,37], and is stronger from close peers such as friends [38]. At the same time, online communication networks do not serve as effective channels for performance transmission. Students tend to segregate in online networks based on their performance and this prevents the diffusion of achievements through online ties [18,19].

Summarizing, the majority of studies demonstrate that social networks are effective channels for the performance diffusion. It was shown that achievements spread well within friendship networks, while other types of ties (e.g. online relationships) do not serve as channels for the performance transmission. In this paper, we examine the diffusion of academic achievements in two distinct social networks: friendship and study assistance. We demonstrate that, despite the significant overlap in these networks, they exhibit different patterns of behavior transmission.

### Data collection

We analyze the longitudinal data on friendship and study assistance networks and GPA of a first-year student cohort of the Economics department in one of the leading Russian universities in 2013–2014 academic year. In this university, students are randomly assigned by the administration to different study groups of up to 30 students. Lectures are usually delivered to all the cohort simultaneously while seminar classes are delivered to each study group separately. In the first year, most of the courses are obligatory. Therefore, students have a limited possibility to form networks with students from other groups, programs, or year cohorts. The academic year consists of four modules of two or three months. At the end of each module, students take final tests and exams. The grading system is at a 10-point scale where a higher score indicates a higher level of academic achievement. The course grade is the weighted average of midterm and final exams, homework, essays, and other academic activities during the course. The sample consists of 31% males and 69% females.

The data for this study was gathered from two sources: the longitudinal student questionnaire survey (3 waves during the first academic year: October 2013, February 2014, and June 2014) and the university administrative database. In total, our dataset consists of 117 students that took part in at least two surveys with up to 700 connections between them in total. The detailed over-time aspect of the networks gives us a rich dataset of links of diverse nature. The sample can be considered representative to student cohorts in selective universities.

In the questionnaire survey, we ask students about their connections within their cohort. The questions were formulated in the following way:

1. Please indicate the classmates with whom you spend most of your time together;
2. Please indicate the classmates whom you ask for help with your studies.

There were no limitations in the number of nominations. Additionally, students were asked to indicate those classmates whom they knew before the admission to the university. We also gather information about students’ study-group affiliation from the administrative database. In total, we have four different network types: friendship, study assistance, knowing each other before studies, and being in the same study group.

From an administrative database of the university, we gather data about student performance (grade point average, or GPA at the end of the first year) that is measured on a scale from 0 to 10. We transform the data on performance from continuous to categorical scale and distinguish four performance groups based on the grading system of the university: high performing students (their GPA is equal or higher than 8), medium high performing students (with GPA from 6 to 8), medium low performing students (with GPA from 4 to 6) and low performing students (with GPA lower than 4).

It is important to mention that the information about individual student grades is publicly available in this university. This is common in some Russian universities but very different from educational systems in the European Union and the US. In Russian universities, grades are often publicly announced by teachers to the class. In the studied university, final grades are additionally published online on the university website. This creates a specific case when students know about the grades of each other and can coordinate their social connections depending on this information.

**Fig 1:**
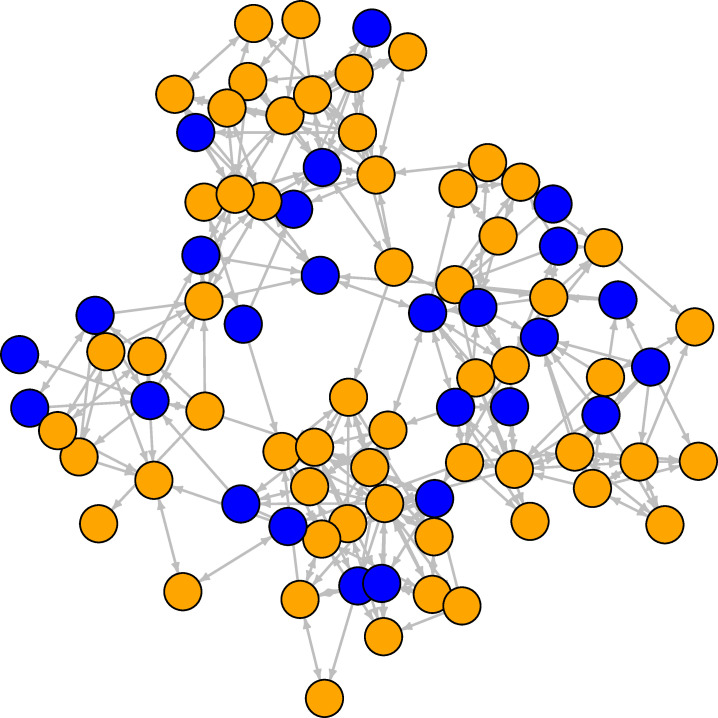
Student friendship network at the first wave. The orange nodes are females, the blue nodes are males. Ties are directed friendship connections between students.

**Fig 2:**
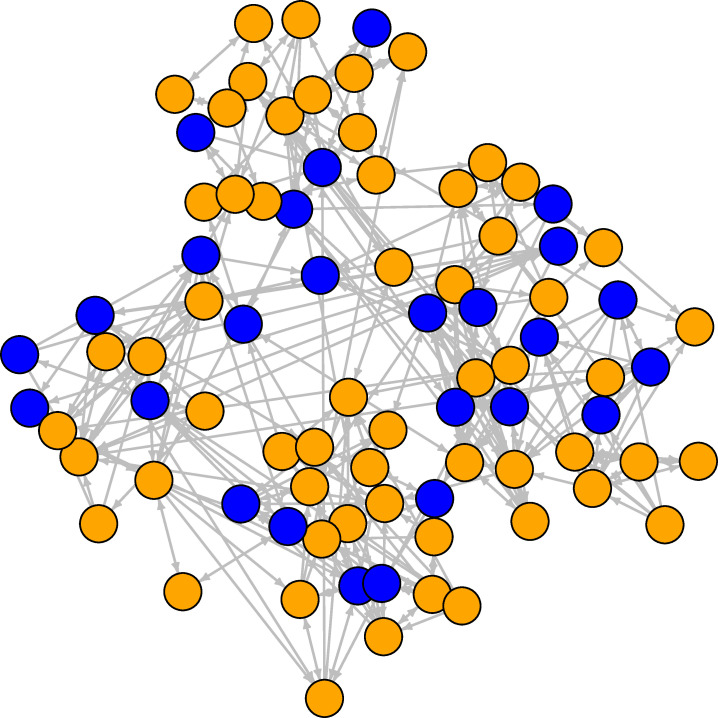
Student friendship network at the second wave. The orange nodes are females, the blue nodes are males. Ties are directed friendship connections between students.

**Fig 3:**
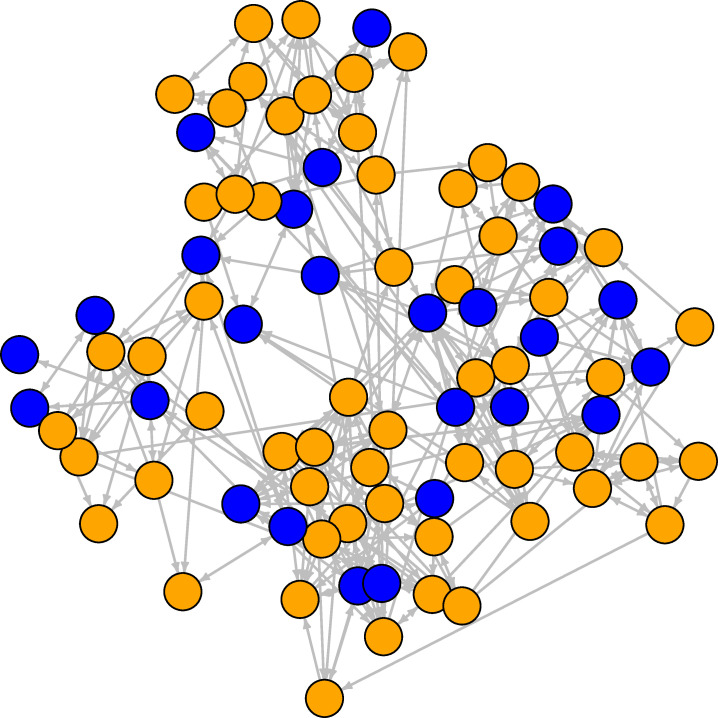
Student friendship network at the third wave. The orange nodes are females, the blue nodes are males. Ties are directed friendship connections between students.

**Fig 4:**
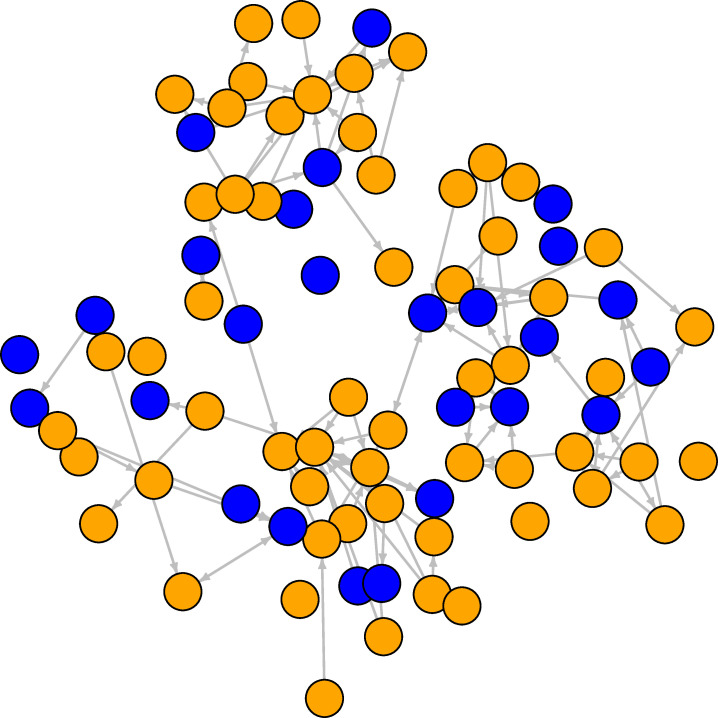
Student study assistance network at the first wave. The orange nodes are females, the blue nodes are males. Ties are directed study assistance connections between students.

**Fig 5:**
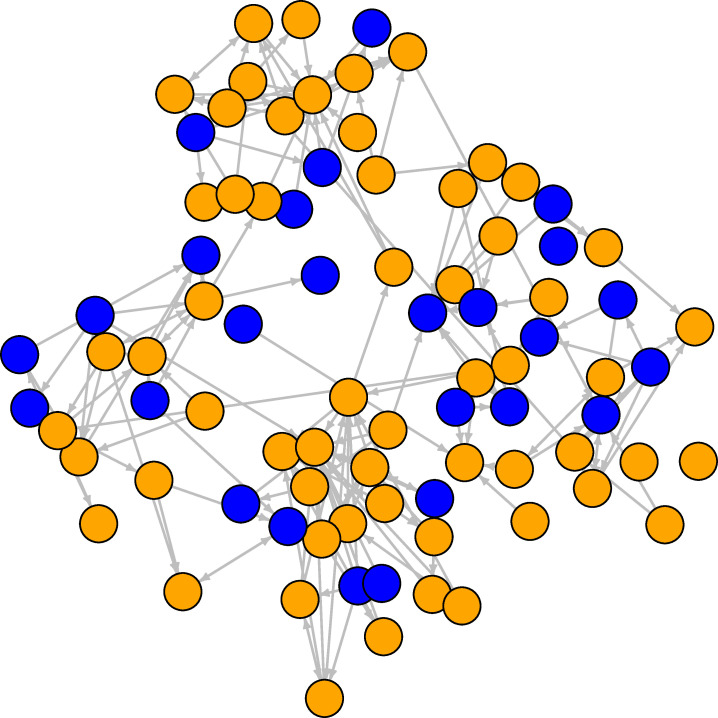
Student study assistance network at the second wave. The orange nodes are females, the blue nodes are males. Ties are directed study assistance connections between students.

**Fig 6:**
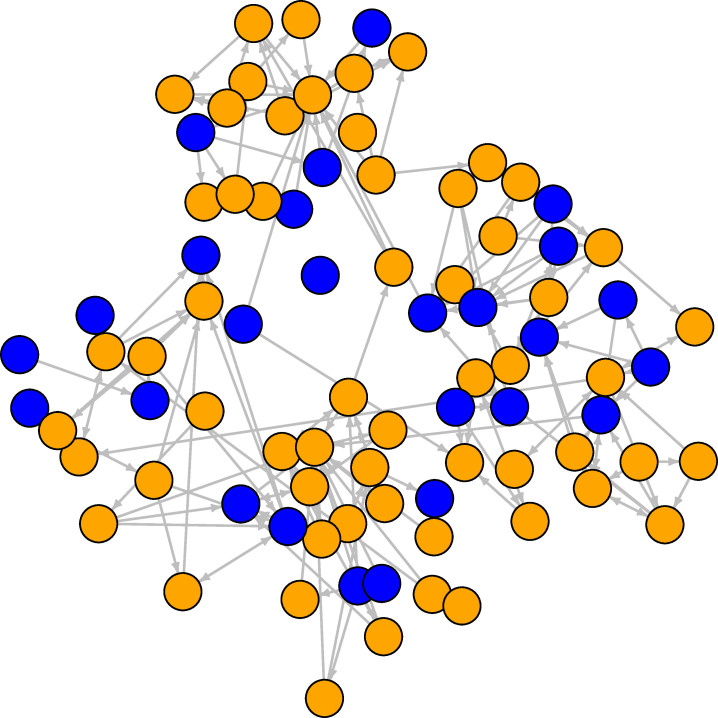
Student study assistance network at the third wave. The orange nodes are females, the blue nodes are males. Ties are directed study assistance connections between students.

Individuals who did not participate in the questionnaire survey were excluded from the analysis (14 individuals, 10.7% of the sample). These missing data were not treated in a special way. We followed the recommendations (40), suggesting that ‘up to 10% missing data will usually not give many difficulties or distortions, provided missingness is indeed non-informative’. Data collection procedures are described in the “Data collection” section in S1 File. The descriptive statistics of the sample are presented in the SI (“Case description” section and Tables 1–3 in S1 File). The network visualizations are presented in Figs 1–6.

## Method

### Stochastic actor-oriented models

Standard statistical techniques such as regression models are not applicable for the analysis of social networks due to the interdependence of network observations [39]. Therefore, we apply a stochastic actor-oriented model (SAOM) that allows to reveal the coevolution of network properties and behavior of actors [25,40]. This dynamic model is widely used for studying the joint evolution of social networks, actor attributes, and separating the processes of social selection and social influence. In total, we estimate two models: the first model estimates the coevolution of friendship network and academic performance, the second one estimates the coevolution of study assistance network and academic performance.

The SAOM’s underlying principles are the following. Firstly, network and behavior changes are modeled as Markov processes which means that the network state at time *t* depends only on the (t-1) network state. Secondly, SAOM is grounded on the methodological approach of structural individualism. It is assumed that all actors are fully informed about the network structure and attributes of all other network participants. Thirdly, time moves continuously and all the macro-changes of the network structure are modeled as a result of the sequence of the corresponding micro-changes. This means that an actor, at each point in time, can either change one of the outgoing ties or modify his or her behavior. The last principle is crucial for the separation of the social selection and social influence processes.

There are four sub-components of the coevolution of network and behavior: network rate function, network objective function, behavior rate function, and behavior objective function [25,40]. The rate functions represent the expected frequencies per unit of time with which actors get an opportunity to make network and/or behavioral micro-changes (40). The objective functions are the primary determinants of the probabilities of changes. The probabilities of the network and/or behavior change are higher if the values of the objective functions for the network/behavior are higher [25,40].

The objective functions for the network (Eq 1) and behavior change (Eq 2) are calculated as a linear combination of a set of components called effects:

$$f_{i}(\beta ,x)=\sum_{k}\beta_{k}S_{ki}(x)$$ (Eq 1)

$$f_{i}^{z}(\beta ,x,z)=\sum_{k}\beta_{k}^{z}S_{ki}^{z}(x,z)$$ (Eq 2)

where s_ki_(x) are the analytical functions (also called effects) that describe the network tendencies (40); β_kz_S_ki_^z^(x, z) are functions that depend on the behavior of the focal actor i, but also on the behavior of his or her network partners and a network position [22]; β_k_ and β_k_^z^ are statistical parameters that show the significance of the effects. SAOM coefficients are interpreted as logistic regression coefficients. Parameters are unstandardized, therefore, the estimates for different parameters are not directly comparable. During the modeling, SAOM allows the inclusion of endogenous and exogenous covariates.

As endogenous variables, we include in our models network density, reciprocity, popularity, activity, transitivity, 3-cycles, transitive reciprocated triplets, and betweenness [40]. Density and reciprocity show the tendency of students to form any ties and to form mutual ties. Transitivity, 3-cycles, transitive reciprocated triplets, and betweenness measure a propensity of students to form triadic connections with their peers. Popularity and activity are included to control the tendency of actors to receive many ties from others and to nominate a large number of actors. To control for social selection, we include the selection effect based on academic achievement. It shows whether students with similar levels of academic achievements tend to form connections with each other. We also controlled for the tendency of students with high grades to increase their popularity and activity over time.

To test the presence of social influence, we include the effect of performance assimilation. It shows whether students tend to assimilate the academic achievement levels of their peers. In addition, we controlled for the propensity of students with high levels of popularity and activity to change their academic performance.

In the model construction we follow the general network modeling requirements necessary for SAOM [40].

### Ethics statement

All research protocols were approved by the HSE (Higher School of Economics) Committee on Interuniversity Surveys and Ethical Assess of Empirical Research. All human subjects gave their informed verbal consent prior to their participation in this research, and adequate steps were taken to protect participants’ confidentiality.

## Results

Table 1 presents the modeling results of two separate models. In the first, we model the coevolution of friendship network and academic performance. In the second one, we model the coevolution of study assistance network and academic performance.

**Table 1:** Results on the coevolution of friendship and study assistance social networks and academic achievements.

Parameter	Estimate (SE) for friendship network	Estimate (SE) for study assistance network
1. Rate parameter 1	16.996 (1.502) ***	7.292 (1.082) ***
2. Rate parameter 2	15.311 (1.577) ***	7.032 (0.821) ***
**Control network effects**		
3. Density	-2.260 (0.120) ***	-3.328 (0.263) ***
4. Reciprocity	1.690 (0.107) ***	0.854 (0.228) ***
5. Popularity	-0.029 (0.011) *	0.055 (0.019) **
6. Activity	0.026 (0.010) **	0.002 (0.025)
**Triadic effects**		
7. Transitivity	0.330 (0.028) ***	0.3941 (0.0886) ***
8. 3-cycles	-0.175 (0.06) **	-0.261 (0.319)
9. Transitive reciprocated triplets	-0.120 (0.054) *	0.014 (0.361)
10. Betweenness	-0.131 (0.028) ***	-0.265 (0.070) ***
**Ties in exogenous networks**		
11. Acquaintance before enrollment	0.9389 (0.1317) ***	0.713 (0.184) ***
12. Studying in the same group	0.687 (0.073) ***	1.187 (0.128) ***
13.1. Tie in study assistance network	0.043 (0.011) ***	
13.2. Tie in friendship network		0.959 (0.111) ***
**Gender effects**		
14. Gender of alter (1—Male)	0.110 (0.065)	0.003 (0.120)
15. Gender of ego (1—Male)	0.161 (0.063) *	0.005 (0.142)
16. Gender similarity	0.240 (0.058) ***	0.239 (0.101) *
**Academic performance effects**		
17. Performance of alter	0.132 (0.057) *	0.836 (0.235) ***
18. Performance of ego	0.219 (0.053) ***	0.057 (0.254)
19. Performance similarity (selection)	0.412 (0.219)	1.994 (0.814) *
**Behavior dynamics effects**		
20. Rate parameter 1	0.550 (0.111) ***	0.636 (0.142) ***
21. Rate parameter 2	1.132 (0.251) ***	1.500 (0.305) ***
22. Linear shape effect	1.134 (0.629)	-0.585 (0.423)
23. Quadratic shape effect	0.452 (0.447)	-0.529 (0.217) *
24. Performance assimilation (influence)	7.797 (3.890) *	4.622 (2.990)
25. Indegree (popularity) effect on performance	0.041 (0.099)	0.393 (0.186) *
26. Outdegree (activity) effect on performance	-0.129 (0.126)	-0.163 (0.178)

Significance codes

*** P < 0.001

** P < 0.01

* P < 0.05. The models converged according to the t-ratios for convergence and the overall maximum convergence ratio criteria suggested in (40). Goodness of fit is adequate for all models.

### Academic performance diffusion

Social influence [effect 24] is positive and significant in the friendship social network. This means that the academic performance of students tends to become similar to the performance of their friends. In other words, academic achievements diffuse through friendship ties. In the study assistance network, however, social influence is not present. This indicates that students do not assimilate the performance of their study assistants; this network channel does not propagate the spread of academic achievements.

Positive indegree effect [effect 25] suggests that students who are often asked for help increase their performance over time. The non-significant estimates of the linear and quadratic shape parameters [effects 22 and 23] for friendship indicate that the influence of peers sufficiently explains the performance dynamics [15]. The negative effect of the quadratic shape parameter [effects 23] for the study assistance network shows the convergence of the academic performance to unimodal distribution [15].

### Academic performance impact on network evolution

The effect of performance selection [effect 19] is positive for the study assistance network. It suggests that students with similar levels of academic achievements tend to ask each other for help. The effect of social selection in the friendship network is not significant. This means that students do not have a preference to befriend students with similar academic achievements.

Positive estimates for the performance of alter [effect 17] in both social networks suggest that individuals with high performance are popular in friendship and study assistance networks. Positive effect for the performance of ego [effect 18] for friendship network shows that high performing students tend to create friendship connections.

### Gender and network evolution

We find the presence of gender homophily [effect 16] in both friendship and study assistance social networks. Students tend to create friendship and study assistance connections with individuals with the same gender. Positive effect of ego for males [effect 15] in the friendship network suggests that males tend to nominate more friends.

### Evolution of network structures

The network control effects [effects 3, 4, 7, 8, 9, and 10] that were included in the models show expected signs and significance scores, as in most student social networks [25]. The negative density for both networks [effect 3] indicates that actors tend to create ties that are embedded in complex local configurations. Positive reciprocity effect [effect 4] shows that individuals tend to form mutual connections, both in friendship and study assistance networks. In the friendship network, the combination of positive transitivity [effect 7] and negative 3-cycles [effect 8] reveals the presence of a local hierarchy. Negative transitive reciprocated triplets effect [effect 9] shows that transitivity is less important for friendship ties when reciprocity is present (and vice versa) [41]. The combination of negative betweenness [effect 10] and positive transitivity [effect 7] in both networks demonstrate that individuals do not seek for brokerage positions and do not want to connect peers from different network communities and study groups.

Positive activity effect [effect 6] in friendship network indicates that students with many ties tend to create new friendship relationships. Positive effect of popularity [effect 5] in study assistance network suggests that individuals ask for help those students, who are often asked for help by others. In friendship network this effect is negative, which means that students do not tend to befriend popular individuals, i.e. those who already have a lot of friends.

In both networks rate parameters are larger in the first period rather than in the second, indicating that the tie formation stabilizes over time.

### Ties in exogenous networks

The modeling results also show that students tend to create friendship and study assistance ties with individuals they knew before the enrollment [effect 11] and individuals from the same study group [effect 12]. Also, students tend to create friendship connections with their study assistants [effect 13.1] and they seek for study assistance from their friends [effect 13.2].

### Time heterogeneity

We conducted the time heterogeneity test for both network models [40]. This test is used to examine whether the parameter values β_k_ of the objective function are constant over the periods of observation. We find the time heterogeneity in models. In both networks, parameters such as betweenness, acquaintance before enrollment, popularity and activity of the high performing individuals are heterogeneous. In the friendship network, there is also time heterogeneity for gender of alter and ego, performance social selection and influence. In the study assistance network, we find time heterogeneity for studying in the same group, gender of ego, and performance of ego.

The cases of previous acquaintance or being in the same study group can be explained by the nature of these types of ties. For instance, the acquaintance before enrollment can play a significant role at the beginning of studies, while after several months’ students will tend to expand their networks and will not seek connections with individuals they knew before studies. The same explanation can be used for the case of being in the same study group. At the beginning of studies, students will form ties within their study groups but later they will tend to expand their network and form ties with other group members. Differences in time heterogeneity of academic achievements may be related to the decreased statistical power of these effects between different models.

### Social selection and influence for different achievement groups

The effects of academic performance on network evolution processes may be understood in details by considering all the performance-related effects simultaneously [40]. In Table 2, we present log-odds for the performance selection within different achievement groups. The higher the estimate, the higher the probability of a study assistance tie formation between students from different performance groups. Table 2 shows that there is a significant tendency toward selection of high-performing individuals as study assistants, and this tendency is present among all groups of students.

**Table 2:** Total performance effects on log-odds of study assistance selection.

	Alter, performance				
Ego, performance		Low performing	Medium low performing	Medium high performing	High performing
	Low performing	-0.811	-0.474	-0.136	0.202
	Medium low performing	-1.2538	0.0817	0.419	0.757
	Medium high performing	-1.694	-0.360	0.975	1.312
	High performing	-2.135	-0.801	0.533	1.868

Similarly, in Table 3 we present precise estimates for the social influence process for all achievement groups. Each row of the table corresponds to a given average behavior of the friends of an ego. Values in the row show the relative ‘attractiveness’ of the different potential values of ego’s behavior. Maximum diagonal value indicates that for each value of the average friends’ behavior the actor ‘prefers’ to have the same behavior as all these friends (40). This shows that individuals tend to assimilate their friends’ performance.

**Table 3:** Estimates for the social influence for different achievement groups.

	Alter, performance				
Ego, performance		Low performing	Medium low performing	Medium high performing	High performing
	Low performing	1.289	-0.660	-2.609	-4.559
	Medium low performing	-0.474	1.475	-0.474	-2.423
	Medium high performing	-1.333	0.617	2.566	0.617
	High performing	-1.287	0.663	2.612	4.561

## Discussion

In this paper we explore the academic performance diffusion through two social networks of different natures: friendship and study assistance. We empirically confirm that educational outcomes of students are diffused in different ways within friendship and study assistance networks. Ties in the friendship network transmit academic achievements, while ties in the study assistance network do not. The absence of the social influence process along the presence of social selection in the study assistance network may suggest the presence of social segregation based on performance [19]. This can be related to the high competitiveness of the university environment under the study. We expect that some students are highly motivated to receive higher grades and prefer to invest time and effort in their high academic results rather than help their less academically successful peers. Our findings demonstrate that the efficacy of academic achievements diffusion is determined by the nature of the social network. It was established that social integration in the classroom is positively associated with the higher academic performance of students [17,19]. Here we claim that it is extremely important to integrate individuals specifically in the network of informal friendship interactions and motivate them to create connections with higher-performing students.

These findings support the idea that the nature of social relationships is crucial for the transmission of specific types of information and behavior in social networks. Close friendship relationships serve as effective channels for the spread of various complex behaviors, including very costly behavior types such as health behavior [1]. Academic performance is one of the examples of these behavior types that are not easily transmittable. In contrast, the instrumental study assistance ties do not produce the propagation of academic achievements from successful students to their lower-performing peers. To sum up, we show that costly and complex behavior (such as academic achievement) diffuses more effectively in the network of strong close connections such as friendship. These findings contribute to the current debates on behavior propagation in social networks and propose new insights on factors that impact the success of behavior transmission.

This study has several limitations. First, we analyze social networks of first-year students. This time frame, when students start their educational path at an undergraduate level, receives a lot of attention in the literature [17,19,42] due to the fast speed of social tie formation. At the same time, it would be beneficial to investigate the diffusion of academic achievements through social networks along the full period of studies. Second, we examine only two types of social relations, however, the spectrum of social ties that can serve as channels of the performance diffusion is much wider. It is a potential avenue for future studies to estimate the efficacy of other types of social networks such as cooperation, competition, romantic relationships, and negative ties on the process of academic achievement diffusion. The data on some of these networks is difficult to collect (e.g., negative relationships) due to the high sensitivity of studied relationships but these types of ties can be nevertheless significant for behavior transmission. In the time of the COVID-19 pandemic and after it, it is also extremely important to examine the effect of online networks on academic performance transmission because online interaction remains the only communication channel for students.

Our empirical findings have several policy implications. Academic achievements are one of the key components of financial success and individual well-being [43,44], that makes the performance increase is one of the main goals of the educational system. However individual achievements are quite stable and largely driven by heritable factors [45] which make interventions aimed at the academic performance growth highly complex and difficult to implement. One of the possible mechanisms of performance increase is social influence, as we show in this paper. Teachers can pay additional attention to the development of informal friendship relationships between students with various performance levels during classes. It can be reached by group work assignments, in which group membership is defined by the teachers and is not based on the personal preferences of students. Long-term group assignments such as working on a research project together can stimulate students from different achievement groups to develop friendship ties with each other. The creation of recreation and open spaces within the university building can also give additional options for students with distinct performance to meet, interact, and form friendship ties. The combination of these actions would help students to build and sustain their informal networks, which, in turn, serve as key channels of the academic performance diffusion and lead to a positive behavior change.

## Supporting information

S1 File (DOCX)
