# Supplementary material for: How academic achievement spreads: The role of distinct social networks in academic performance diffusion
Source: PLoS One. 2020 Jul 27;15(7):e0236737. doi: 10.1371/journal.pone.0236737 (PMC7384671; doi:10.1371/journal.pone.0236737)
Supplement: S1 File — (DOCX) [file pone.0236737.s001.docx]

## Case description

In this study, we use the data about social networks and individual characteristics of first-year Economics students that studied in 2013–14 in a selective Russian university. We analyze social connections and performance in their first-year when students do not know each other well and their friendship ties are still under construction.

Overall, the number of students in the cohort is 131 and they are distributed in 5 study groups. We analyzed data about networks of 117 students (90% of the sample), who took part in at least two surveys. 89% students participated in the first wave, 79% in the second, and 76% in the third.

Less than half of students (41.5%) pay tuition fees, while others are tuition-free. The majority of respondents (96%) do not work. Only a small fraction of students (4%) have sporadic work or freelance. The major part of respondents (72.7%) lives with their parents or relatives, 24.0% rent apartments, and a few students (3.3%) live in the dormitory. There is almost no variance in students’ socioeconomic status. The families of the major part of students (87.0%) have enough money for food and clothes but have difficulties with large purchases. A small fraction of respondents report that their families are well off (6.5%) and even smaller fraction of participants’ families can afford expensive purchases (2.2%). Only 4.3% of students report that the wages of their parents cover their daily costs but they cannot easily afford buying clothes.

## Data collection

The student survey was conducted in the following manner. The questionnaires were distributed to students 10-15 minutes before the end of their classes and there was enough time to finish a survey before the break. The participants filled in the questionnaires in the classroom. We conducted the survey during the courses that are obligatory for attendance in order to obtain the data about the majority of students. If some students were absent, we additionally followed them up during the following two weeks and asked to fill in the questionnaire. There were no exclusion criteria for students to participate in the study; the entire cohort was included in the survey. In network studies, it is crucial to minimize the amount of the missing data and gather the information about the full network. For this reason, we tried to gather data about the majority of students.

## Descriptive statistics

**Table 1. Descriptive statistics for the student performance groups.**

| Parameter | First wave | Second wave | Third wave |
| --- | --- | --- | --- |
| “High performing” fraction | 7% | 7% | 14% |
| “Medium high performing” fraction | 35% | 36% | 55% |
| “Medium low performing” fraction | 55% | 46% | 26% |
| “Low performing” fraction | 3% | 11% | 4% |

**Table 2. Friendship network descriptive statistics**

| Network parameter | First wave | Second wave | Third wave |
| --- | --- | --- | --- |
| Number of students | 117 | 117 | 117 |
| Number of links among students | 715 | 662 | 557 |
| Density | 0.05 | 0.05 | 0.04 |
| Reciprocity | 0.63 | 0.60 | 0.51 |
| Transitivity | 0.42 | 0.37 | 0.35 |
| Jaccard coefficient | - | 0.35 | 0.32 |

**Table 3. Study assistance network descriptive statistics**

| Network parameter | First wave | Second wave | Third wave |
| --- | --- | --- | --- |
| Number of students | 117 | 117 | 117 |
| Number of links among students | 226 | 267 | 248 |
| Density | 0.02 | 0.02 | 0.02 |
| Reciprocity | 0.24 | 0.23 | 0.19 |
| Transitivity | 0.29 | 0.28 | 0.27 |
| Jaccard coefficient | - | 0.28 | 0.26 |
